# Literature based species occurrence data of birds of northeast India

**DOI:** 10.3897/zookeys.150.2002

**Published:** 2011-11-28

**Authors:** Sujit Narwade, Mohit Kalra, Rajkumar Jagdish, Divya Varier, Sagar Satpute, Noor Khan, Gautam Talukdar, V. B. Mathur, Karthikeyan Vasudevan, Dinesh Singh Pundir, Vishwas Chavan, Rajesh Sood

**Affiliations:** 1 Bombay Natural History Society (BNHS), Shaheed Bhagatsingh Road, 400001, Mumbai, India; 2 Wildlife Institute of India (WII), Post Box No 18, Chandrabani, 248001, Dehradun, India; 3 Global Biodiversity Information Facility (GBIF), Universitetsparken 15, DK 2100, Copenhagen, Denmark

**Keywords:** India, northeast Himalaya, Assam, Arunachal Pradesh, Bihar, Manipur, Meghalaya, Mizoram, Nagaland, Sikkim, Tripura, West Bengal, Uttar Pradesh, *Journal of the Bombay Natural History Society*, BNHS, Animalia, Chordata, Aves

## Abstract

The northeast region of India is one of the world’s most significant biodiversity hotspots. One of the richest bird areas in India, it is an important route for migratory birds and home to many endemic bird species. This paper describes a literature-based dataset of species occurrences of birds of northeast India. The occurrence records documented in the dataset are distributed across eleven states of India, viz.: Arunachal Pradesh, Assam, Bihar, Manipur, Meghalaya, Mizoram, Nagaland, Sikkim, Tripura, Uttar Pradesh and West Bengal. The geospatial scope of the dataset represents 24 to 29 degree North latitude and 78 to 94 degree East longitude, and it comprises over 2400 occurrence records. These records have been collated from scholarly literature published between1915 and 2008, especially from the *Journal of the Bombay Natural History Society* (JBNHS). The temporal scale of the dataset represents bird observations recorded between 1909 and 2007. The dataset has been developed by employing MS Excel. The key elements in the database are scientific name, taxonomic classification, temporal and geospatial details including geo-coordinate precision, data collector, basis of record and primary source of the data record. The temporal and geospatial quality of more than 50% of the data records has been enhanced retrospectively. Where possible, data records are annotated with geospatial coordinate precision to the nearest minute. This dataset is being constantly updated with the addition of new data records, and quality enhancement of documented occurrences. The dataset can be used in species distribution and niche modeling studies. It is planned to expand the scope of the dataset to collate bird species occurrences across the Indian peninsula.

## Data published through GBIF:

http://ibif.gov.in:8080/ipt/resource.do?r=BNHS-NEW

## Taxonomic coverage

**General taxonomic coverage description:** The taxonomic coverage of this dataset spans Class Aves. The highest number of data records are from the family Muscicapidae (560 records), followed by Anatidae (180 records) and Accipitridae (136 records). The families with the least number of records are Hemiprocnidae, Podargidae, Indicatoridae with one data record each.

### Taxonomic ranks

**Kingdom:** Animalia

**Phylum:** Chordata

**Class:** Aves

**Order:** Podicipediformes, Pelecaniformes, Ciconiiformes, Anseriformes, Falconiformes, Galliformes, Gruiformes, Charadriiformes, Columbiformes, Psittaciformes, Cuculiformes, Strigiformes, Caprimulgiformes, Apodiformes, Trogoniformes, Coraciiformes, Piciformes, Passeriformes

**Family:** Podicipedidae, Phalacrocoracidae, Anhingidae, Ardeidae, Ciconiidae, Threskiornithidae, Anatidae, Accipitridae, Pandionidae, Falconidae, Phasianidae, Turnicidae, Gruidae, Rallidae, Otididae, Jacanidae, Rostratulidae, Charadriidae, Scolopacidae, Recurvirostridae, Glareolidae, Laridae, Rhynchopidae, Columbidae, Psittacidae, Cuculidae, Tytonidae, Strigidae, Podargidae, Caprimulgidae, Apodidae, Hemiprocnidae, Trogonidae, Alcedinidae, Meropidae, Coraciidae, Upupidae, Bucerotidae, Capitonidae, Indicatoridae, Picidae, Eurylaimidae, Pittidae, Alaudidae, Hirundinidae, Motacillidae, Campephagidae, Pycnonotidae, Irenidae, Laniidae, Troglodytidae, Prunellidae, Muscicapidae, Aegithalidae, Paridae, Sittidae, Certhiidae, Dicaeidae, Nectariniidae, Zosteropidae, Emberizidae, Fringillidae, Estrildidae, Passeridae, Sturnidae, Oriolidae, Dicruridae, Artamidae, Corvidae.

## Spatial coverage

**General spatial coverage:** This dataset collates species occurrences from northeast India and neighboring regions. The occurrence records collated in the dataset are *Literature-based species occurrence data of birds of North-East India 3* distributed across eleven provinces of India, viz.: Arunachal Pradesh, Assam, Bihar, Manipur, Meghalaya, Mizoram, Nagaland, Sikkim, Tripura, Uttar Pradesh and West Bengal. This region falls within the Himalayan mountain ranges, and spans an area of 234567 sq. km. The region borders with Bangladesh to the south, Bhutan to the west, Myanmar to the east and with China to the north. Minimum and maximum elevations are 2000 meters and 8000 meters above sea level respectively.

**Coordinates:** 24°30'0"N - 28°15'0"N Latitude; 78°22'58.8"E - 93°47'60"E Longitude.

## Temporal coverage

1909–2007.

## Project description

**Title:** This dataset is an outcome of the collaborative work carried out by three projects, viz.:, (a) Environmental Information System (ENVIS) Centre on Avian Ecology, Bombay Natural History Society, sponsored by the Ministry of Environment and Forests, Government of India; (b) Important Bird Areas Programme and Indian Bird Conservation Network (IBA-IBCN), sponsored by the Royal Society for Protection of Birds, United Kingdom; and (c) Impact of Climate Change on the Conservation of Birds, a project supported by the MacArthur Foundation.

**Personnel:** Sujit Narwade (Author, Content Provider, Metadata Provider), Mohit Kalra (Processor), Divya Varier (Custodian Steward/Metadata Provider), Rajkumar Jagdish (Processor), Sagar Satpute  (Custodian Steward), Noor Khan (Custodian Steward), Gautam Talukdar (Publisher), V.B. Mathur (Publisher), Karthik Vasudevan (Publisher), Dinesh Singh Pundir (Publisher), Vishwas Chavan (Metadata Provider/Editor), Rajesh Sood (Metadata Provider/Programmer).

**Funding:** Ministry of Environment and Forests, Government of India, (Funding), the Royal Society for Protection of Birds, United Kingdom (Funding), MacArthur Foundation (Funding), Bombay Natural History Society (Host institution), and Wildlife Institute of India (publishing support).

**Study area descriptions/descriptor:** Northeast India is one of the most significant biodiversity hotspots of the world and among the richest bird zones in India. It is considered as the ‘biological gateway’ for much of India’s fauna, as the Gondwana land first touched this region, during the Tertiary period. The north-eastern region is at the confluence of the Indo-Malayan, Indo-Chinese and Indian biogeographical realms. As a result, it is unique in providing a profusion of habitats that harbor diverse biota with a high degree of endemism (Chatterjee et al. 2006).

**Design description:** The Environmental Information System (ENVIS) Centre at the Bombay Natural History Society (BNHS) is a focal point for collection, collation and dissemination of data on avian ecology in India. The objective of this dataset is to collate avian observations documented in various research publications such as journals, magazines, newsletters, project reports, theses, books and other gray literature. However, the current version of the dataset collates occurrence records reported in research articles published in the *Journal of the Bombay Natural History Society *(JBNHS). The motivation for this approach is because of the dispersed availability of the occurrence records in published literature. Because these data records are documented in several literature sources, it is difficult to access them together, inspite of being in the public domain. Thus, for a potential user it is not possible to access and use them when he/she needs them the most. Another consideration is the quality of these data records, as they are published in peer reviewed literature and their quality is expected to be ‘fit for use’. The data records were entered in a MS-Excel worksheet. The offline version of the dataset maintained by the Bombay Natural History Society is developed employing MS-Access. The key elements about which information is collated in the current version of the dataset includes: scientific name, common name, taxonomic classification, occurrence location, geo-coordinates, precision of geo-coordinates, date of observation, data collector, and primary source of the data record.

The data records were entered in the MS-Excel worksheet. The offline version of the dataset maintained by the Bombay Natural History Society is developed employing the MS-Access. The key elements about which information is collated in the current version of the dataset includes, scientific name, common name, taxonomic classification, occurrence location, geo-coordinates, precision of geo-coordinates, date of observation, data collector, and primary source of the data record.

## Dataset description

**Object name:** Darwin Core Archive literature-based species occurrence data of birds of northeast India

**Character encoding:** UTF-8

**Format name:** Darwin Core Archive format

**Format version:** 1.0

**Distribution:** http://ibif.gov.in:8080/ipt/archive.do?r=BNHS-NEW

**Publication date of data:** 2011-06-30

**Language:** English

**Licenses of use:** by-nc-sa

**Metadata language:** English

**Date of metadata creation:** 2011-06-30

**Hierarchy level:** Dataset

## Additional information

We are most thankful to the Ministry of Environment and Forests (MoEF), Government of India; the Royal Society for the Protection of Birds (RSPB), United Kingdom; and the MacArthur Foundation for financial support. We would like to express our deepest gratitude to Dr. Asad R. Rahmani, Director, BNHS and Principal Investigators of the aforementioned projects.

## Appendix I

Literature-based species occurrence data of birds of North-East India. (doi: 10.3897/zookeys.150.2002.app) File format: XLS

**Explanation note:** This is an Excel spreadsheet of the dataset, available through the Darwin Core Archive format at: http://ibif.gov.in:8080/ipt/resource.do?r=BNHS-NEW

## References

[B1] Abdulali H (1983) Occurrence of the Great Crested Grebe: *Podiceps cristatus* (Linne) at Ranchi. Bihar. J. Bombay Nat. Hist. Soc. 80 (2) 414–415.

[B2] Abdulali H (1954) More notes on Finn’s Baya (*Ploceus megarhynchus*): J. Bombay Nat. Hist. Soc. 52 (2&3): 599–601.

[B3] Abdulali H (1957) Some notes on the plumages of *Centropus sinensis* (Stephens): J. Bombay Nat. Hist. Soc. 54 (1) 183–185.

[B4] Abdulali H (1961) The nesting habits of the eastern race of Finn’s Baya, *Ploceus megarhynchus *Salim Ali Abdulali: J. Bombay Nat. Hist. Soc. 58 (1) 269–270.

[B5] Abdulali H (1964) Notes on Indian birds 1-*Ceyx erithacus rufidorsus* Strickland in the Sikkim Terai, Eastern Himalayas: An addition to the Indian avifauna. J. Bombay Nat. Hist. Soc. 61 (2) 439–440.

[B6] Abdulali H (1965) Behaviour of Lesser Whistling Teal [*Dendrocygna javanica* (Horsfield)] in Alipore zoo Calcutta: J. Bombay Nat. Hist. Soc. 62 (2) 300–301.

[B7] Allen D Holt PI Hornbuckle J (2002) Leaf-presenting as possible courtship behaviour by Pied Falconets *Microhierax melanoleucos*: J. Bombay Nat. Hist. Soc. 99 (3) 518–520.

[B8] Altevogi R Davis TA (1979) Urbanization in nest building of Indian House Crow (*Corvus splendens *Vieillot): J. Bombay Nat. Hist. Soc. 76 (2) 283–290.

[B9] Barua M (2002) Occurrence of the Indian Skimmer *Rhynchops albicollis* Swainson in Assam: J. Bombay Nat. Hist. Soc. 99 (3) 526.

[B10] Baruah M Chettri G Bordoloi P (2004) Sighting of White-bellied Heron *Ardea insignis* Hume in Pobitora wildlife sanctuary: J. Bombay Nat. Hist. Soc. 101 (2) 311.

[B11] Bertram B (1967) Hill Myna *Gracula religiosa* Linnaeus breeding in artificial nests in Garo hills Assam: J. Bombay Nat. Hist. Soc. 64 (2) 369–370.

[B12] Betts FN (1952) Bird nesting on telegraph wires: J. Bombay Nat. Hist. Soc. 51 (1) 271–272.

[B13] Betts FN (1955) Notes on birds of the Subansiri area Assam: J. Bombay Nat. Hist. Soc. 53 (4) 397–414.

[B14] Burley H (1954) Occurrence of the Blacknecked Crane (*Grus nigricollis*) in Indian limits: J. Bombay Nat. Hist. Soc. 52 (2&3): 605–606.

[B15] Burnett JH (1958) Photographing a colony of Egrets (*Bubulcus ibis* and *Egretta garzetta*) in Assam: J. Bombay Nat. Hist. Soc. 55 (3) 565–566.

[B16] Chatterjee S (1995) Occurrence of Albino Lesser Whistling Teal: *Dendrocygna javanica* (Horsfield). J. Bombay Nat. Hist. Soc. 92 (3) 417–418.

[B17] Chatterjee S Mookerjee K Bhattacharya B Banerjee A (1995) Occurrence of Falcated Teal *Anas falcata* Georgi in West Bengal: J. Bombay Nat. Hist. Soc. 92 (2) 262.

[B18] Chattopadhyay S (1978) Occurrence of the Thickbilled Warbler *Phragmaticola aedon**rufescens* Stegmann at Baj Baj West Bengal: J. Bombay Nat. Hist. Soc. 75 (2) 491–492.

[B19] Chattopadhyay S (1980) Egg-bound death of a Purplerumped Sunbird at Baj Baj West Bengal: J. Bombay Nat. Hist. Soc. 77 (2) 333.

[B20] Chattopadhyay S (1980) Observations on parental care of a wounded chick of the Bronze-winged Jacana: *Metopidius indicus* (Latham). J. Bombay Nat. Hist. Soc. 77 (2) 325–326.

[B21] Choudhury A (1991) Purple-rumped Sunbird *Nectarinia zeylonica* (Linn.): A new record for Assam. J. Bombay Nat. Hist. Soc. 88 (1) 114.

[B22] Choudhury A (1992) Addition to the birds of Assam - Blacknecked *Grebe Podiceps nigricollis *Brehm: J. Bombay Nat. Hist. Soc. 89 (2) 245–246

[B23] Choudhury A (1993) Additions to the birds of Assam: White-tailed Sea eagle and Large Sand Plover. J. Bombay Nat. Hist. Soc. 91 (1) 139.

[B24] Choudhury A (1993) On a possible sight records of the Little Gull *Larus minutus* Pallas in Arunachal Pradesh: J. Bombay Nat. Hist. Soc. 90 (2) 290.

[B25] Choudhury A (1998) Common Myna feeding a fledgling Koel: J. Bombay Nat. Hist. Soc. 95 (1) 115.

[B26] Choudhury A (1998) The Bengal Florican *Eupodotis bengalensis* Gmelin 1789 in Dibang valley district of Arunachal Pradesh: J. Bombay Nat. Hist. Soc. 95 (2) 342.

[B27] Choudhury A (2004) Sighting of Wallcreeper *Tichodroma muraria* in Assam and Manipur: J. Bombay Nat. Hist. Soc. 101 (3) 463.

[B28] Choudhury A (2005) Great-tufted Myna *Acridotheres grandis* - An addition to the birds of Meghalaya: J. Bombay Nat. Hist. Soc. 102 (1) 117.

[B29] Choudhury A (2005) Migration of Black-eared or Large Indian Kite *Milvus migrans lineatus* (Gray) from Mongolia to North-eastern India: J. Bombay Nat. Hist. Soc. 102 (2) 229–230.

[B30] Das PK (1965) The Whitecheeked Drongo [*Dicrurus leucophaeus salangensis* Reichenow] : An Addition to the Indian avifauna. J. Bombay Nat. Hist. Soc. 62 (3) 557–558.

[B31] Datta A (2004) Sighting of the Oriental Bay-Owl *Phodilus badius saturatus* in Pakhui Wildlife sanctuary Western Arunachal Pradesh: J. Bombay Nat. Hist. Soc. 101 (1) 156.

[B32] Davis TA (1973) Mud and dung plastering in Baya nests: J. Bombay Nat. Hist. Soc. 70 (1) 57–71.

[B33] Editors (1952) The whimbrel (*Numenius phaeopus*) in Assam: J. Bombay Nat. Hist. Soc. 50 (3) 663.

[B34] Editors BNHS (1958) The Avocet (*Recurvirostra avosetta* Linn.) in Assam: J. Bombay Nat. Hist. Soc. 55 (1) 170.

[B35] Fooks HA (1966) Whistling Teal [*Dendrocygna javanica* (Horsefield)] and other memories of Alipore zoo Calcutta: J. Bombay Nat. Hist. Soc. 63 (1) 200–202.

[B36] Ganguli U (1990) Blackwinged Kite *Elanus caeruleus vociferus* (Latham) at 3650m in Sikkim: J. Bombay Nat. Hist. Soc. 87 (1) 142.

[B37] Ganguli U (1990) Brahminy Duck *Tadorna ferruginea* (Pallas) Breeding in Sikkim: J. Bombay Nat. Hist. Soc. 87 (2) 290.

[B38] Ganguli U (1990) Osprey *Pandion haliaetus* in Sikkim: J. Bombay Nat. Hist. Soc. 87 (2) 291.

[B39] Ganguli-Lachungpa U Lucksom S (1998) Sighting of Hodgson’s Frogmouth *Batrachostomus hodgsoni hodgsoni* (G.R. Gray) from Sikkim: J. Bombay Nat. Hist. Soc. 95 (3) 505.

[B40] Ganguli-Lachungpa U Lucksom S (1998) Western Greyheaded Thrush *Turdus rubrocanus* G.R. Gray in Sikkim: J. Bombay Nat. Hist. Soc. 95 (3) 508–509.

[B41] Ganguli-Lachungpa U (1991) Occurrence of Blacknecked Grebe *Podiceps nigricollis* Brehm: Little Grebe *P. ruficollis* (Pallas) and Goosander *Mergus merganser* Linn. in West Sikkim. J. Bombay Nat. Hist. Soc. 88 (2) 280.

[B42] Ganguli-Lachungpa U (1998) Attempted breeding of the Blacknecked Crane *Grus nigricollis* Przevalski in North Sikkim: J. Bombay Nat. Hist. Soc. 95 (2) 341.

[B43] Ganguli-Lachungpa U (2002) Eurasian Eagle-owl *Bubo bubo tibetanus* Bianchi at 2,100 M in north Sikkim: J. Bombay Nat. Hist. Soc. 99 (2) 305–306.

[B44] Ganguli-Lachungpa U (2003) Common coot *Fulica atra* from Kyongnosla in East Sikkim: J. Bombay Nat. Hist. Soc. 100 (1) 121.

[B45] Ganguly JK (1986) Co-operative feeding of chicks of the Purple-rumped Sunbird (*Nectarinia zeylonica*): J. Bombay Nat. Hist. Soc. 83 (2) 447.

[B46] Gauntlett FM (1971) Durgapur barrage as a waterbird habitat: J. Bombay Nat. Hist. Soc. 68 (3) 619–632.

[B47] Gauntlett FM (1985) The birds of Durgapur and the Damodar valley: J. Bombay Nat. Hist. Soc. 82 (2) 501–539.

[B48] Gee EP (1958) The present status of the Whitewinged Wood Duck *Cairina scutulata* (S. Muller): J. Bombay Nat. Hist. Soc. 55 (3) 569–575.

[B49] George PV (1967) On the occurrence of the Fulvous-brested Woodpecker *Dendrocopos macei* (Vieillot) in Sikkim: J. Bombay Nat. Hist. Soc. 64 (3) 559–560.

[B50] Ghose D Khan S (2005) Albino bulbul at Keibul Lamjao national park, Manipur India: J. Bombay Nat. Hist. Soc. 102 (1) 120–121.

[B51] Green MJB (1986) The birds of the Kedarnath sanctuary Chamoli district Uttar Pradesh: Status and distribution. J. Bombay Nat. Hist. Soc. 83 (3) 603 – 617.

[B52] Gupta KK (1966) Aggressive behaviour of a Spotted Owlet [*Athene brahma* (Temminck)]: J. Bombay Nat. Hist. Soc. 63 (2) 441–442.

[B53] Gupta KK (1995) A note on Baya, *Ploceus philippinus* nesting on Krishnachuda (Delonix regia) tree: J. Bombay Nat. Hist. Soc. 92 (1) 124–125.

[B54] Haribal M Ganguli-Lachungpa (1991) Black Woodpecker *Dryocopus Sp*. In Jaldapara sanctuary West Bengal: J. Bombay Nat. Hist. Soc. 88 (1) 112.

[B55] Harington HH (1915) Notes on Indian Timaliides and their allies: Pt. 4. J. Bombay Nat. Hist. Soc. 23: 614-657

[B56] Holmes JRS (1968) New wintering locality of the Spotted Bush Warbler *Bradypterus thoracicus* (Blyth): J. Bombay Nat. Hist. Soc. 65 (3) 779–780.

[B57] Hopper CD (1958) Occurrence of the Baikal Teal *Nettion formosum* (Georgi) in Assam: J. Bombay Nat. Hist. Soc. 55 (2) 359–360.

[B58] Hussain SA (1984)*Hypsipetes madagascariensis sinensis* (La touch): A first record for India. J. Bombay Nat. Hist. Soc. 81 (1) 195–196.

[B59] Jackson PFR (1974) Goliath Heron in the Sundarbans West Bengal: J. Bombay Nat. Hist. Soc. 71 (3) 608–609.

[B60] Javed S (1995) Hare in the diet of White-eyed Buzzard Eagle *Butastur teesa* (Franklin): J. Bombay Nat. Hist. Soc. 92 (1) 119.

[B61] Jha A (2001) Competition between Jungle Myna *Acridotheres fuscus* and Lesser Goldenbacked Woodpecker *Dinopium benghalense* for a nest hole: J. Bombay Nat. Hist. Soc. 98 (1) 115.

[B62] Jha A (2002) More evidence of Red-vented Bulbul *Pycnonotus cafer* feeding on House Gecko *Hemidactylus flaviviridis*: J. Bombay Nat. Hist. Soc. 99 (1) 118.

[B63] Jha S (1994) An Albino Myna *Acridotheres tristis* (Linnaeus): J. Bombay Nat. Hist. Soc. 91 (3) 455.

[B64] Jha S (2000) A note on the feeding of Lesser Coucal (*Centropus toulou*): J. Bombay Nat. Hist. Soc. 97 (1) 144.

[B65] Jha S (2002) Attempted feeding by a Shikra *Accipiter badius*, family Accipitridae on Buffstriped Keelback *Amphiesma stolata* family Colubridae: J. Bombay Nat. Hist. Soc. 99 (2) 298.

[B66] Kalita SK (2000) Competition for food between a Garden Lizard *Calotes versicolor* (Daudin) and a Magpie Robin *Copsychus saularis* Linn: J. Bombay Nat. Hist. Soc. 97 (3) 431

[B67] Kumar RS (2003) Ring recovery from Great Cormorants *Phalacrocorax carbo* in India: J. Bombay Nat. Hist. Soc. 100 (3) 621–624.

[B68] Kumar RS (2004) Common Starling *Sturnus vulgaris* in Arunachal Pradesh India: J. Bombay Nat. Hist. Soc. 101 (2) 320.

[B69] Lahkar B (2000) Pallas’s fishing Eagle *Haliaeetus leucoryphus* (Pallas) pirates fish from an Otter *Lutra lutra* (Linn.): J. Bombay Nat. Hist. Soc. 97 (3) 425.

[B70] Lahkar K Phukan MP (2007) Wintering range extension of White-throated Bushchat *Saxicola insignis* (Gray) in India: J. Bombay Nat. Hist. Soc. 104 (3) 348–349.

[B71] Lamba BS (1981) A queer nesting site of Bank Myna, *Acridotheres ginginianus*: J. Bombay Nat. Hist. Soc. 78 (3) 605–606.

[B72] Law SC (1952) Occurrence of the Smew [*Mergellus albellus* (Linn.)] in West Bengal: J. Bombay Nat. Hist. Soc. 51 (2) 508–509.

[B73] Lister MD (1952) Secondary song of some Indian birds: J. Bombay Nat. Hist. Soc. 51 (3) 699–706

[B74] Lister MD (1954) A contribution to the ornithology of the Darjeeling area: J. Bombay Nat. Hist. Soc. 52 (1) 20–68

[B75] Madge SC (1984) First Indian record of Chaffinch (*Fringilla coelebs*): J. Bombay Nat. Hist. Soc. 81 (3) 702–703.

[B76] Mansion P (1967) The whistling Teal [*Dendrocygna javanica* (Horsfield)] in the Calcutta environs: J. Bombay Nat. Hist. Soc. 64 (3) 558–559.

[B77] Matthews WH (1952) Breeding of *Rallina euryzonoides**nigrolineata* (Gray) in the Darjeeling district: J. Bombay Nat. Hist. Soc. 51 (3) 742–743.

[B78] Meinertzhagen R (1952) A new bird for India - *Montifringilla davidiana potanini* (Sushkin): J. Bombay Nat. Hist. Soc. 51 (1) 273–274.

[B79] Mohan D Chellam R (1990) New call record of Greenbreasted Pitta *Pitta sordida* (P.L.S. Muller) in Dehra Dun Uttar Pradesh: J. Bombay Nat. Hist. Soc. 87 (3) 453.

[B80] Mohan D Rai ND Singh AP (1992) Longtailed Duck or Old Squaw *Clangula hyemalis* (Linn.) in Dehra Dun Uttar Pradesh: J. Bombay Nat. Hist. Soc. 89 (2) 247.

[B81] Mukherjee AK (1969) Food-habits of water-birds of the Sundarban: 24-Parganas district West Bengal India. J. Bombay Nat. Hist. Soc. 66 (2) 345–360.

[B82] Mukherjee AK (1971) Food-Habits of water-birds of the Sundarban: 24-Parganas district West Bengal India – III. J. Bombay Nat. Hist. Soc. 68 (1) 690 – 716.

[B83] Mukherjee AK (1974) Food-habits of water-birds of the Sundarban: 24 Parganas district West Bengal India – IV. J. Bombay Nat. Hist. Soc. 71 (2) 188–200.

[B84] Mukherjee AK (1975) Food-habits of waterbirds of the Sundarban: 24 Parganas district West Bengal India – V. J. Bombay Nat. Hist. Soc. 72 (2) 422–447.

[B85] Mukherjee AK (1975) The Sundarban of India and its biota: J. Bombay Nat. Hist. Soc. 72 (1) 1–20.

[B86] Mukherjee AK (1976) Food-habits of water-birds of the Sundarban: 24 Parganas district West Bengal India – VI. J. Bombay Nat. Hist. Soc. 73 (2) 482- 486.

[B87] Mukhopadhyay A (1980) Some observations on the biology of the Openbill Stork: *Anastomus oscitans* (Boddaert) in Southern Bengal. J. Bombay Nat. Hist. Soc. 77 (1) 133–137.

[B88] Mukhrjee AK (1971) Food-habits of water-birds of the Sundarban: 24-Parganas district West Bengal India – II. J. Bombay Nat. Hist. Soc. 68 (1) 37–64.

[B89] Murthy S (1954) An intelligent Myna: J. Bombay Nat. Hist. Soc. 52 (2&3): 598.

[B90] Naoroji R Sangha HS Barua M (2005) The Lesser Kestrel *Falco naumanni* and Amur Falcon *Falco amurensis* in the Garo hills: Meghalaya India. J. Bombay Nat. Hist. Soc. 102 (1) 103–105.

[B91] Narayan G Rosalind L (1991) New record of the Pied Harrier *Circus melanoleucos* (Pennant) breeding in Assam Duars, with a brief review of its distribution: J. Bombay Nat. Hist. Soc. 88 (1) 30–34.

[B92] Narayan G & Rosalind L (1994) Wintering and time extension of Hodgson’s Bush Chat *Saxicola insignis* (Gray) in India: J. Bombay Nat. Hist. Soc. 94 (3) 572–573.

[B93] Rahmani AR Sankaran R (1990) An unusual nesting site of the Sunbird: J. Bombay Nat. Hist. Soc. 87 (1) 148–149.

[B94] Rahmani AR (1981) Large Racket-tailed Drongo and Common Babbler: J. Bombay Nat. Hist. Soc. 78 (2) 380.

[B95] Rahmani AR (1981) Narora reservoir U.P - A potential bird sanctuary: J. Bombay Nat. Hist. Soc. 78 (1) 88–92.

[B96] Rahmani AR (1991) Status of the Bengal Florican *Houbaropsis bengalensis* in India: J. Bombay Nat. Hist. Soc. 88 (3) 349–375.

[B97] Rao KR Zoramthanga R (1978) On the phenomenon of nocturnal flights of some resident birds at Lunglei: Mizoram N.E. India. J. Bombay Nat. Hist. Soc. 75 (3) 927–928.

[B98] Rao P Murlidharan S (1989) Unusual feeding behaviour in the Adjutant Stork *Leptoptilos dubius *(Gmelin): J. Bombay Nat. Hist. Soc. 86 (1) 97.

[B99] Rao P Grubh RB Muralidharan S (1989) Range extension of Eurasian Griffon Vulture *Gyps fulvus*: J. Bombay Nat. Hist. Soc. 86 (2) 240–241.

[B100] Rasool TJ (1984) Some observations on Natural Cheer Pheasant, *Catreus wallichii*: Population at Mukteshwar reserve forest. Kumaon, Nainital UP. J. Bombay Nat. Hist. Soc. 81 (2) 469–471.

[B101] Raza R (1993) Sighting of Black Bulbul *Hypsipetes madagascariensis* (P.L.S. Muller) in Gaya, Bihar: J. Bombay Nat. Hist. Soc. 90 (2) 291.

[B102] Ripley SD (1952) A collection of birds from the Naga hills: J. Bombay Nat. Hist. Soc. 50 (3) 475–514.

[B103] Ripley SD (1980) A new species, and a new subspecies of bird from trip district Arunachal Pradesh and comments on the subspecies of *Stachyris nigriceps* Blyth: J. Bombay Nat. Hist. Soc. 77 (1) 1–5.

[B104] Ritschard M Taschler A (2008) A recent observation of White-headed *Duck Oxyura leucocephala* at Gajaldoba barrage West Bengal India: J. Bombay Nat. Hist. Soc. 105 (1) 95.

[B105] Ritschard M Logtmeijer P Taschler A (2008) Two observations of Malayan Night-heron *Gorsachius melanophus* from West Bengal, India: J. Bombay Nat. Hist. Soc. 105 (1) 97–98.

[B106] Saha BC Mukherjee AK (1978) Notes on the food of the Blackheaded Munia and the Spotted Munia in South Kamrup district Western Assam (India): J. Bombay Nat. Hist. Soc. 75 (1) 221–224.

[B107] Saha SS (1976) Occurrence of Finn’s Baya (*Ploceus megarhynchus* Hume) in Dirrang district: Assam. J. Bombay Nat. Hist. Soc. 73 (3) 527–529.

[B108] Saha SS (1980) Blacknecked Crane in Bhutan and Arunachal Pradesh - A survey report for January- February 1978: J. Bombay Nat. Hist. Soc. 77 (2) 326–328.

[B109] Saha SS George PV Ghosal DK Mookerjee HP Poddar AK Ghose RK Das PK Gogate VG Biswas B (1971) Notes on some interesting birds from the salt lakes, near Calcutta: J. Bombay Nat. Hist. Soc. 68 (2) 455–457.

[B110] Sankaran R (1989) Range extension of Yellowbellied Wren-Warbler *Prinia flaviventris*: J. Bombay Nat. Hist. Soc. 86 (3) 451.

[B111] Sankaran R Rahmani AR Ganguli-Lachungpa U (1992) The distribution and status of the Lesser Florican *Sypheotides indica* (J.F. Miller) in the Indian subcontinent: J. Bombay Nat. Hist. Soc. 89 (2) 156–179.

[B112] Sarma P Barua M Menon V (1997) Orangebilled Jungle Mynah and Hodgson’s Bushchat in Kaziranga National Park: J. Bombay Nat. Hist. Soc. 94 (1) 156–157.

[B113] Satheesan SM (1990) Biometrics and food of some doves of the genus *Streptopelia*: J. Bombay Nat. Hist. Soc. 87 (3) 452–453.

[B114] Satheesan SM (1993) Extension of range of the Kashmir Roller (Blue Jay) *Coracias garrulus* to Gorakhpur Uttar Pradesh: J. Bombay Nat. Hist. Soc. 90 (1) 95.

[B115] Sendall D (1952) Occurrence of the Avocet (*Recurvirostra avocetta* Linn.) in Assam: J. Bombay Nat. Hist. Soc. 50 (4) 947.

[B116] Sengupta S (1973) Significance of communal roosting in the Common Myna [*Acridotheres tristis *(Linn.)]: J. Bombay Nat. Hist. Soc. 70 (1) 204–206.

[B117] Sengupta S (1975) Further note on the pair formation of the Common Myna, *Acridotheres tristis*: J. Bombay Nat. Hist. Soc. 72 (3) 856–857.

[B118] Sengupta SN (1969) Nest protection by the Indian House Crow (*Corvus splendens* Linnaeus): J. Bombay Nat. Hist. Soc. 66 (2) 377–378.

[B119] Sharma A Zockler C (2008) First record of Caspian Gulls *Larus cachinnans* in the Indian Sunderbans delta: J. Bombay Nat. Hist. Soc. 105 (1) 93–94.

[B120] Sharma A (2008) Record of Large congregation of Large Whistling-Duck *Dendrocygna bicolor *in the Purbasthali-ganges islets Burdwan district West Bengal: J. Bombay Nat. Hist. Soc. 105 (1) 97.

[B121] Sharma A (2008) Sighting of Indian Skimmer *Rhynchops albicollis* (Swainson) in the Purbasthali-ganges islets Burdwan district West Bengal: J. Bombay Nat. Hist. Soc. 105 (1) 92–93.

[B122] Singh KS (1998) Aerial display of Rufous Turtle Dove *Streptopelia orientalis agricola* Tickell near Nambol bazaar Manipur: J. Bombay Nat. Hist. Soc. 95 (1) 114.

[B123] Singh P (1992) Spotted Longtailed Wren-babbler *Spelaeornis troglodytoides* (Verreaux) in Arunachal Pradesh: J. Bombay Nat. Hist. Soc. 89 (3) 376.

[B124] Singh P (1995) Occurrence of Swamp Partridge *Francolinus gularis* (Temminck) in Arunachal Pradesh: J. Bombay Nat. Hist. Soc. 92 (3) 419.

[B125] Singha H Rahmani AR Coulter MC Javed S (2003) Breeding behaviour of the Greater Adjutant-stork *Leptotilos dubius* in Assam, India: J. Bombay Nat. Hist. Soc. 100 (1) 9–26.

[B126] Singha H Karim R Rahmani AR (1999)*Menopon gallinae* infesting Greater Adjutant Stork *Leptoptilos dubius* at Nagaon Assam: J. Bombay Nat. Hist. Soc. 96 (1) 137–138.

[B127] Sivakumar S Prakash V, (2004) Cat snake *Boiga trigonata* in diet of Jerdon’s *Baza Aviceda jerdoni*: J. Bombay Nat. Hist. Soc. 101 (3) 445–446.

[B128] Taylor JN (1954) Occurrence of Bronzecapped or Falcated Teal (*Eunetta falcata*) near Calcutta: J. Bombay Nat. Hist. Soc. 52 (2&3): 607.

[B129] Yahya SA (1981) Golden Oriole (*Oriolus oriolus*) feeding a fledgling Cuckoo (*Cuculus sp.):* J. Bombay Nat. Hist. Soc. 78 (2) 379–380.

[B130] Islam MZ Rahmani AR (2004) Important bird areas in India: Priority sites for conservation. Indian Bird Conservation Network, BNHS and BirdLife International. Pp xviii + 1133.

[B131] Chatterjee S Saikia A Dutta P Ghosh D Pangging G Goswami AK (2006) Biodiversity significance of north east India: WWF-India. New Delhi. Pp 71.

